# Adherence to Mediterranean-style diet and risk of sepsis in the REasons for Geographic and Racial Differences in Stroke (REGARDS) cohort

**DOI:** 10.1017/S0007114518002866

**Published:** 2018-10-30

**Authors:** Marquita S. Gray, Henry E. Wang, Kimberly D. Martin, John P. Donnelly, Orlando M. Gutiérrez, James M. Shikany, Suzanne E. Judd

**Affiliations:** 1 Department of Biostatistics, University of Alabama at Birmingham (UAB), Birmingham, AL 35294, USA; 2 Department of Emergency Medicine, University of Texas Health Science Center at Houston, Houston, TK 77030, USA; 3 Department of Epidemiology, UAB, Birmingham, AL 35294, USA; 4 Department of Emergency Medicine, UAB, Birmingham, AL 35233, USA; 5 Division of Nephrology, UAB School of Medicine, Birmingham, AL 35233, USA; 6 Division of Preventive Medicine, UAB School of Medicine, Birmingham, AL 35205, USA

**Keywords:** Mediterranean-style diet, Dietary intake, Sepsis, Infection, Prevention

## Abstract

Sepsis – syndrome of infection complicated by organ dysfunction – is responsible for over 750 000 hospitalisations and 200 000 deaths in the USA annually. Despite potential nutritional benefits, the association of diet and sepsis is unknown. Therefore, we sought to determine the association between adherence to a Mediterranean-style diet (Med-style diet) and long-term risk of sepsis in the REasons for Geographic Differences in Stroke (REGARDS) cohort. We analysed data from REGARDS, a population-based cohort of 30 239 community-dwelling adults age ≥45 years. We determined dietary patterns from a baseline FFQ. We defined Med-style diet as a high consumption of fruit, vegetables, legumes, fish, cereal and low consumption of meat, dairy products, fat and alcohol categorising participants into Med-style diet tertiles (low: 0–3, moderate: 4–5, high: 6–9). We defined sepsis events as hospital admission for serious infection and at least two systematic inflammatory response syndrome criteria. We used Cox proportional hazard models to determine the association between Med-style diet tertiles and first sepsis events, adjusting for socio-demographics, lifestyle factors, and co-morbidities. We included 21 256 participants with complete dietary data. Dietary patterns were: low Med-style diet 32⋅0%, moderate Med-style diet 42⋅1% and high Med-style diet 26⋅0%. There were f 109 (5⋅2%) first sepsis events. High Med-style diet was independently associated with sepsis risk; low Med-style diet referent, moderate Med-style diet adjusted hazard ratio (HR) 0⋅93 (95% CI 0⋅81, 1⋅08), high Med-style diet adjusted HR=0⋅74 (95% CI 0⋅61, 0⋅88). High Med-style diet adherence is associated with lower risk of sepsis. Dietary modification may potentially provide an option for reducing sepsis risk.

Microbial infections including pneumonia, kidney infection, cellulitis and meningitis can trigger sepsis^(1)^, which is a life-threatening organ dysfunction resulting from a dysregulated host response to infection^(2)^. Sepsis is responsible for approximately 200 000 deaths in the USA annually^(3)^. In 2014, there were over 750 000 hospitalisations for sepsis in the USA^(4)^.

Research reveals that diet may play an integral role in influencing sepsis risk. Previous studies have shown that high-fat diets fed to mice are associated with increased mortality, organ injury, susceptibility to sepsis and disturbance in innate immune functions^(5–7)^. Although this study was done in mice and may not be applicable for humans, a disturbance in innate immune functions can affect the body’s ability to combat microbial infections^(8)^, which as mentioned above can trigger sepsis. A study, in humans, demonstrated that a Southern-style diet – high consumption of processed meats, fried foods and sugar-sweetened beverages – is associated with an increased risk of sepsis^(9)^. Other studies have shown that a greater adherence to a Western diet – high consumption of red meats, fat/dairy productions and refined grains – is associated with higher levels of biomarkers of endothelial and inflammation dysfunction^(10–12)^, which are both implicated in the biological pathway of sepsis^(13–15)^. These two human studies show that a Southern and Western dietary pattern are associated with a higher risk of sepsis. Despite these findings, there is a lack of research on the association between the Mediterranean-style diet (Med-style diet) and risk of sepsis.

The concept of a Mediterranean diet originated in the Mediterranean basin after the second world war^(16)^. It is a social practice that is based on knowledge, traditions, fishing, conservation, preparation, as well as culture^(16)^. A traditional Mediterranean diet consists of a high consumption of plant foods, high olive oil intake as the primary source of monounsaturated fat, low saturated fat intake with limited meat and dairy products consumption, and moderate fish and alcohol intake^(17)^. Med-style diet has been reported as a model for healthy eating secondary to its contributions to a more favourable health status and overall quality of life^(18,19)^, as well as its beneficial roles in reducing CVD and chronic degenerative disease occurrence^(18,20)^. One study concluded that lower adherence to a Med-style diet was associated with higher risk of stroke^(21)^. In contrast, another study showed that higher adherence to a Med-style diet was associated with a 30% risk reduction in CVD^(22)^ and lower incidence of major CVD events, breast cancer and diabetes^(23)^. Since Med-style diet is associated with decreased risk of stroke and prior stroke is associated with greater risk of sepsis^(24)^, we would expect to observe a decrease in risk of sepsis within the high Med-style diet adherence group. In this study, we seek to examine the association between adherence to Med-style diet and risk of sepsis in community-dwelling participants of the REasons for Geographic and Racial Differences in Stroke (REGARDS) study.

## Methods

### Study design and participants

REGARDS is a national, prospective, longitudinal study that enrolled 30 239 individuals from January 2003 to October 2007. Briefly, this study investigated black-white and regional differences in stroke incidence with an oversampling of black and white individuals. This sampling method included 30% of participants from the stroke belt (North Carolina, South Carolina, Georgia, Tennessee, Mississippi, Alabama, Louisiana and Arkansas), 20% from the stroke buckle region (the coastal plain of North Carolina, South Carolina and Georgia), and 50% from other regions in the continental USA. Participants were at least 45 years of age at baseline and contacted twice a year via telephone calls. Detailed descriptions of the REGARDS cohort are published else-where^(25–28)^. The REGARDS-sepsis study identified participants with infection and potentially reversible tisk factors for sepsis at the individual and community level. REGARDS participants with data anomalies or without plausible energy intakes/incomplete dietaiy data were excluded from the cunent analysis. The institutional review boards of all participating institutions approved the study and all participants provided written consent.

### Measures

#### Primary exposure.

We used the Block 98 FFQ (NutritionQuest) to estimate usual dietary intake over the past year. To calculate the Med-style diet score, nine food groups were identified: (i) vegetables, (ii) fruits, (iii) legumes, (iv) cereals (which includes bread, pasta and rice), (v) fish, (vi) meat, (vii) dairy products, (viii) fat intake and (ix) alcohol intake^(21)^. Secondly, we regressed energy intake (kj) to calculate the derived residuals of daily intake (g) for seven of the nine food groups (vegetables, fruits, legumes, cereals, fish, meat and dairy products)^(21)^. Individuals received a value of 1 under these conditions: (i) for consumption above the median for each beneficial component (fruit, legumes, cereal, vegetables and fish) and (ii) for consumption below the median for each detrimental component (meat and dairy products)^(21)^. For the eighth food category (fat intake), we used a ratio (monounsaturated fats to saturated fats) of the individual’s daily consumption to calculate the median consumption separately for each sex^(21)^. Those individuals with a ratio equal to or above the median calculated received a score of 1^(21)^. For alcohol intake, individuals with moderate consumption (1–7 drinks/week for women and 1–14 drinks/week for men) received a score of 1^(21)^. The median intake of these components for males and females are shown in online Supplementary Table S1, which is based on Trichopolou’s proposed method^(29)^.

#### Primary outcome.

Sepsis events were ascertained via medical record review from 5 February 2003 to 31 December 2012 and is described elsewhere^(24)^. In brief, this process was conducted in strictly structured manner. Two independently trained research assistants collected and reviewed medical records for all hospitalisations and emergency department visits. Two abstractors independently reviewed all relevant clinical impressions documented in physician notes to confirm the presence of a serious infection during the initial hospital visit. Then these abstractors identified physiologic and laboratory parameters needed to fulfill the sepsis criteria below^(30)^: Sepsis was defined as an admission to the hospital following serious infection and the existence of a minimum of two ‘systemic inflammatory response syndrome’ criteria during the first 28 h of hospital admission: (a) temperature (<36 or >38°C), (b) heart rate >90 beats per min, (c) respiratory rate >20 breaths/min or partial pressure of carbon dioxide (PaCO_2_) <32 mmHg and (d) leucocyte count (<4000 or >12 000 cells/mm^3^). When abstractors disagreed, additional physician-level review was utilised as needed.

#### Secondary outcome.

Our secondary outcome was severe sepsis, which, due to advances in sepsis pathobiology^(31)^, has been differentiated from less severe sepsis^(31)^. We used the established sequential organ failure assessment (SOFA) criteria^(32)^ to identify admitted patients who had a SOFA score of at least two points, which we defined as a severe sepsis event. This score assesses organ dysfunction in six organ systems (respiration, coagulation, liver, cardiovascular, central nervous system and renal) and thresholds for SOFA score calculation are described elsewhere^(33)^.

#### Demographic and lifestyle factors.

REGARDS used a combination of computer-assisted telephone interviews and a home visit follow-up roughly 3–4 weeks post interview to collect baseline data variable information. During the home visit, blood, urine, blood pressure, electrocardiogram, prescription medication and anthropometric data were collected.

Participants were given self-administered questionnaires – including the FFQ – to gather information and return by mail. REGARDS collected information on age, sex, race, region of residence, income, education, smoking status, energy intake (kJ), sedentary behaviour and co-morbid conditions (see online Supplementary Table S2 for detailed description of each variable)^(25–28,34)^.

#### Statistical analysis

We used *χ*^2^ and Kruskal–Wallis tests to compare socio-demographic characteristics across the distribution of Med-style diet scores. Individuals with implausible energy intakes or incomplete dietary data were excluded from analysis. The Med-style diet score was calculated as the sum of the nine food categories (range, 0–9), with a higher score indicating higher adherence to the Med-style diet. Then, we categorised Med-style diet score into low, moderate and high using Med-style diet score tertiles (Med-style diet score, 0–3, 4–5 and 6–9, respectively)^(35–37^, which yielded comparable results to previous studies that evaluated Med-style diet with other health outcomes^(21,35–37)^, with low Med-style diet group as the reference group, as previously described^(21)^. We also compared included *v*. excluded participants. We performed a Cox proportional hazard model analysis adjusted for covariates, which included sociodemographic (age, race, sex, education, income and geographic region), lifestyle factors (smoking status and sedentary behaviour), and co-morbid conditions (diabetes, hypertension, stroke, atrial fibrillation and obesity status) to assess the association between Med-style diet and sepsis outcomes. We used the SOFA criteria to determine the association between Med-style diet and severe sepsis. Based on sepsis and severe sepsis definitions, sensitivity analyses were used to evaluate Med-style diet as a continuous variable. All analyses were performed using SAS version 9.4 (SAS Institute).

## Results

**Fig. 1.**
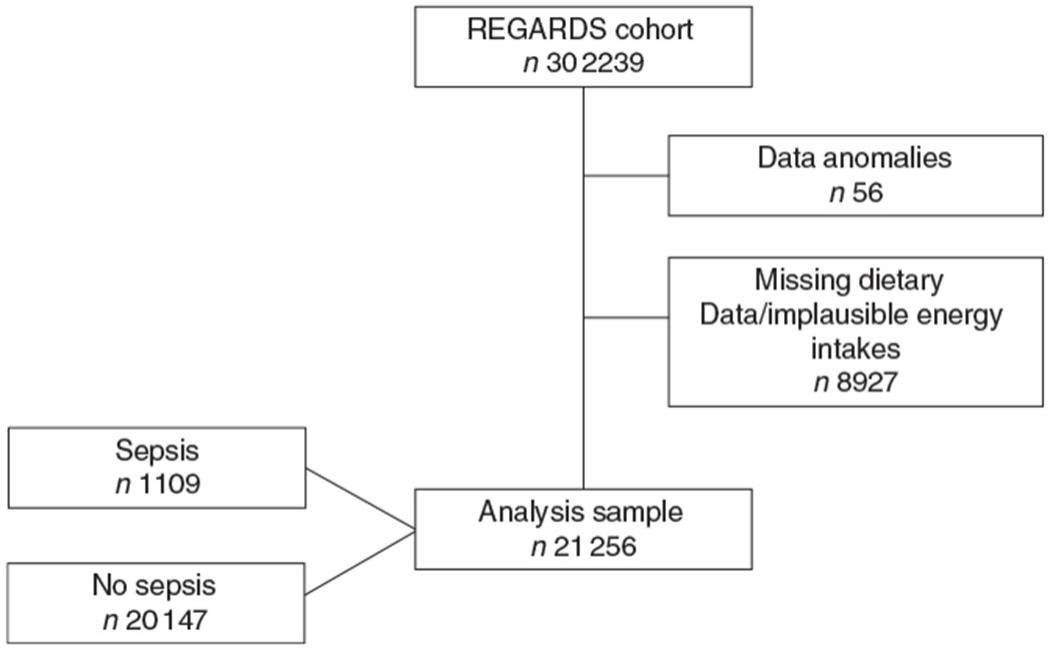
Participant selection. REGARDS, REasons for Geographic and Racial Differences in Stroke.

REGARDS collected information for 30 239 individuals. After excluding those with data anomalies (*n* 56) and with missing dietary data/implausible energy intakes (*n* 8927), a sample of 21256 (70⋅3%) individuals were included in the present analyses (Fig. 1). The median for age was 64⋅0 (Interquartile range 13⋅0) years, 33⋅4% were black (*n* 7099), 56⋅0% were females (*n* 11 905) and 43⋅7% were from the stroke-belt region (*n* 9286). The Med-style diet score ranged from 0 to 9 with an approximately normal distribution, with 26⋅0% of participants (*n* 5519) having a score of 6–9, 42⋅1% of participants (*n* 8940) having a score of 4 or 5 and 32⋅0% of participants (*n* 6797) having a score of 0–3-The mean Med-style diet score was 4⋅4 (sd 1⋅7). Demographic characteristics and lifestyle factors of participants with low, moderate and high Med-style diet are presented in Table 1. Med-style diet score was greater among black race, male sex, older individuals, with greater educational attainment and residents of the non-belt, and was lower among current smokers, those who were obese and lived a sedentary lifestyle, had a lower energy intake, and had a history of diabetes, hypertension and stroke.

We did not observe differences in age or energy intake among individuals who were excluded *v*. included individuals. However, we did observe statistically significant differences for excluded individuals, who were more likely to be lower income, black males with less than high school education who resided in the non-belt region, were obese, lived a sedentary lifestyle, had history of diabetes and stroke, and were current smokers (not shown).

During a mean follow up period of 6⋅4 (sd 2⋅2) years, incident sepsis was identified in 1109 (5⋅2%) participants. Results of our incremental Cox proportional hazard model analysis is shown in Table 2. As compared with low Med-style diet adherence, there were no statistically significant associations of moderate Med-style diet adherence with risk of sepsis in unadjusted or fully adjusted models (hazard ratio (HR) for moderate *v*. low Med-style diet=0⋅88; 95% CI 0⋅77, 1⋅00; HR = 0⋅93; 95% CI 0⋅81, 1⋅08, respectively). Compared with low adherence to Med-style diet, higher adherence to Med-style diet was associated with lower risk of sepsis (HR for high *v*. low Med-style diet=0⋅66; 95% CI 0⋅56, 0⋅78), in the unadjusted model. After adjustment for demographic and socioeconomic factors (model I, model II and model III), this association remained statistically significant (HR=0⋅62; 95% CI 0⋅53, 0⋅74; HR=0⋅70; 95% CI 0⋅59, 0⋅82 and HR=0⋅72; 95% CI 0⋅60, 0⋅86, respectively). In the fully adjusted model (model IV), high adherence to Med-style diet remained associated with lower risk of sepsis (HR for high *v*. low Med-style diet=0⋅74; 95% CI 0⋅61, 0⋅88).

Moderate and high adherence to Med-style diet was associated with a lower risk of severe sepsis in the fully adjusted model (HR for moderate *v*. low Med-style diet=0⋅82; 95% CI 0⋅68, 0⋅97 and HR for high *v*. low Med-style diet=0⋅65; 95% CI 0⋅52, 0⋅81; respectively, table not shown). As a sensitivity analysis, Med-style diet score was evaluated as a continuous variable in a Cox proportional hazards model. In the fully adjusted model, a 1-point increase in Med-style diet score was independently associated with a 6% lower risk of sepsis (95% CI 3, 10). Independent of covariates in the fully adjusted model, a 1-point increase in Med-style diet score was associated with a 9% lower risk of severe sepsis (95% CI 5, 14).

## Discussion

After adjusting for multiple potential confounders in the REGARDS cohort - a large population-based sample of US black and white adults, individuals within the high Med-style diet group had a 30% lower risk of sepsis.

Med-style diet and risk of sepsis has been understudied; therefore, we are unable to compare our results to past studies. However, greater adherence to a Southern-style dietary pattern - high in eggs/egg dishes, sugar-sweetened beverages, fried foods, processed meats and red meat - has been associated with greater sepsis risk^(9)^. This Southern-style pattern contains very different foods from those of the Med-style diet. Studies have also shown that Westernised diets are associated with some key mediators of sepsis, which includes inflammation and endothelial cell activation^(10–12)^, whereas a healthier diet with high consumption of fruits and vegetables was associated with the opposite outcome^(38,39)^. Since the Med-style diet score is indicative of a healthy diet pattern in contrast to a Western or Southern diet pattern, our findings are congruent with those described above.

Our secondary analysis revealed a 18% lower risk of severe sepsis for the moderate Med-style diet adherence group and a 35% lower risk of severe sepsis for the high Med-style diet adherence group, in the fully adjusted model. Based on six organ systems, the SOFA score can capture organ dysfunction^(31)^. A sepsis-SOFA score of 2 or more is associated with a 10% excess in overall in-hospital mortality^(31)^. Considering the vulnerability of this population and severity of organ dysfunction, we would expect to observe a potentially greater benefit of moderate as well as high adherence to Med-style diet.

There are some limitations to consider that can affect the generalisability of our findings. Reliance on FFQ data can have some methodological issues - selection bias - and underestimation of total energy intake, which can result in mis-classification^(40)^. However, in our study, we examined participants based on a continuum of intakes of foods association with Med-style diet, which did not involve analysing the absolute energy intakes of each individual. Underestimation of food intake attenuates relative risk of a disease, which is secondary to nutrition literacy, education, socioeconomic status, availability of certain foods, and access to certain foods^(41–45)^. Secondly, the Med-style diet score was assessed using only baseline data. Therefore, we were not able to measure changes in diet at follow-up, which could potentially affect the reported associations. Generalisability of findings is limited because REGARDS includes only black and white individuals. However, the REGARDS cohort is a national cohort study that has an oversampling of blacks and whites from the USA stroke belt region, which is an area where sepsis rates are higher than the any other part of the USA^(28^).

We acknowledged that all strategies of retrospective chart review are subjects to numerous forms of bias. However, identification of sepsis events was performed in a strictly structured manner. Initially, 1349 medical records were reviewed and excellent inter-rater agreement was shown for presence of serious infection (*κ*=0⋅92) and sepsis (*κ*=0⋅90)^(24)^. Therefore, we believe that this system was actually more objective than expert clinician assessment, as it broke down the classification of sepsis to its component items (presence of serious infection plus at least two abnormalities in temperature, heart rate, respiratory rate or leucocyte count).

Patients were given instructions on how to complete their FFQ and mailed these questionnaires to the study centre, which resulted in available FFQ data for 70⋅4% of participants. As mentioned earlier, there were no appreciable differences in age or energy intake among the excluded *v*. included participants; however, there were statistically significant differences in sex, race, region of residence, income, education, sedentary behaviour and smoking status, which is similar to other studies that examined REGARDS participants and Med-style diet^(21,37)^. The included participants were more likely to be white, female, have higher education and income. They were also less likely than excluded participants to be a smoker or to live a sedentary lifestyle. The included participants appear to have been healthier than the excluded participants. Participation rates among REGARDS participants favourably compare with other studies. In terms of bias, we would have to assume that the reason for an individual dropping out of a study could change the association between diet and disease process. Fortunately, for longitudinal cohort studies, most declines in participation rate are not likely to have a substantial impact on associations between the exposure and outcome^(46)^. Therefore, we suggest that socio-demographic differences in participation rates unlikely affect the reported associations.

In conclusion, high adherence to Med-style diet was associated with a lower risk of sepsis. The moderate and high Med-style diet adherence group was associated with risk reduction of severe sepsis. Our findings suggest an association between Med-style diet and risk of sepsis. Targeted efforts to increase consumption of fruits, vegetables, legumes, fish and cereal with low consumption of meat, dairy products, fat, and moderate alcohol intake could potentially serve as an intervention for reducing risk of sepsis. A randomised control trial showed that a Med-style diet - supplemented with extra-virgin olive oil or nuts, along with high consumption of fruits, vegetables, legumes, white meat, fish and moderate alcohol intake - reduced the incidence of major cardiovascular events^(47)^. This reiterates the potential benefits of a Med-style diet intervention in reducing risk of sepsis. To our knowledge, this is the first study to assess an association between Med-style diet and risk of sepsis as well as severe sepsis in community-dwelling participants. Therefore, future studies should further assess the potential benefits of a Med-style diet in reducing risk of sepsis using the SOFA criteria and assess any differences in Med-style diet over time.

## Supplementary Material

Supplementary tables

## Figures and Tables

**Table 1.** Description of baseline characteristics across tertiles of Mediterranean-style diet (Med-style diet) scores for included study participants* (Numbers and percentages; medians and interquartile ranges (IQR))

	Med-style diet score						
	High, 6–9 (*n* 5519)		Moderate, 4–5 (*n* 8940)		Low, 0–3 (*n* 6797)		
Variables	*n*	%	*n*	%	*n*	%	*P*
Age (years)							<0⋅0001
Median	65		65		63		
IQR	13		14		13		
<65 years	1139	9⋅8	6480	55⋅8	3988	34⋅4	<0⋅0001
Sex							<0⋅0001
Male	2631	28⋅1	3893	41⋅6	2827	30⋅2	
Female	2888	24⋅3	5047	42⋅4	3970	33⋅4	
Race							<0⋅0001
Black	1924	27⋅1	3056	43⋅1	2119	29⋅9	
White	3595	25⋅4	5884	41⋅6	4678	33⋅0	
Region							<0⋅0001
Stroke belt	1761	24⋅1	3101	42⋅4	2445	33⋅5	
Buckle	1108	23⋅8	2034	43⋅6	1521	32⋅6	
Non-belt	2650	28⋅5	3805	41⋅0	2831	30⋅5	
Income							<0⋅0001
<$20k	642	19⋅1	1394	41⋅5	1323	39⋅4	
$20k-$34k	1216	23⋅7	2160	42⋅1	1751	34⋅2	
$35k-$74k	1843	27⋅8	2784	41⋅9	2015	30⋅3	
$75k and above	1207	33⋅2	1539	42⋅3	893	24⋅5	
Refused	611	24⋅6	1063	42⋅7	815	32⋅7	
Education							<0⋅0001
Less than high school	374	18⋅3	807	39⋅5	861	42⋅2	
High school graduate	1082	19⋅9	2305	42⋅5	2040	37⋅6	
Some college	1440	24⋅8	2378	40⋅9	1997	34⋅3	
College graduate and above	2622	32⋅9	3447	43⋅3	1895	23⋅8	
Lifestyle factors							
Current smoker	464	16⋅1	1126	39⋅0	1295	44⋅9	<0⋅0001
Sedentary behaviour	417	15⋅5	1116	41⋅4	1162	43⋅1	<0⋅0001
Energy intake (kJ)							<0⋅0001
Median	6880		6555		6468		
IQR	3609		3706		3742		
Co-morbidities							
Diabetes	963	17⋅5	1851	20⋅8	1430	21⋅1	<0⋅0001
Hypertension	3040	55⋅1	5095	57⋅0	3915	57⋅6	0⋅015
Stroke	238	4⋅3	486	5⋅5	410	6⋅1	<0⋅0001
Atrial fibrillation	452	8⋅4	775	8⋅8	574	8⋅7	0⋅598
Obese	1663	30⋅3	3269	36⋅8	2749	40⋅8	<0⋅0001

* Row percentages may not add to 100 due to rounding.

**Table 2.** Sepsis according to tertiles of Mediterranean-style diet (MeD) score* (Hazard ratios (HR) and 95% confidence intervals)

		Moderate (Med-style diet score, 4–5)		High (Med-style diet score, 6–9)	
Association	Low (MeD score, 0–3)	HR	95% CI	HR	95% CI
Crude	Ref.	0⋅88	0⋅77, 1⋅00	0⋅66	0⋅56, 0⋅78
Model I	Ref.	0⋅84	0⋅74, 0⋅96	0⋅62	0⋅53, 0⋅74
Model II	Ref.	0⋅89	0⋅78, 1⋅02	0⋅70	0⋅59, 0⋅82
Model III	Ref.	0⋅92	0⋅80, 1⋅06	0⋅72	0⋅60, 0⋅86
Model IV	Ref.	0⋅93	0⋅81, 1⋅08	0⋅74	0⋅61, 0⋅88

Ref., reference.

* Model I adjusts for age, race, sex and region. Model II adjusts for model I plus income and education. Model III adjusts for model II plus smoking status, sedentary behaviour and energy intake. Model IV adjusts for model II plus diabetes, hypertension, stroke and obesity status.

## Abbreviations:

HR: hazard ratio
Med-style diet: Mediterranean-style diet
REGARDS: REasons for Geographic and Racial Differences in Stroke
SOFA: sequential organ failure assessment
