# Supplementary material for: Adherence to Mediterranean-style diet and risk of sepsis in the REasons for Geographic and Racial Differences in Stroke (REGARDS) cohort
Source: Br J Nutr. Author manuscript; Available in PMC 2019 Sep 20. (PMC6753832; doi:10.1017/S0007114518002866)
Supplement: Supplementary tables [file NIHMS1050312-supplement-Supplementary_tables.docx]

# APPENDIX 1

| **Table 1. Daily dietary, energy, and alcohol Intake by gender and Med-style diet score group** | | | | | | | | |
| --- | --- | --- | --- | --- | --- | --- | --- | --- |
| Dietary Variable |  | Men^1,2^(n=9351) | | |  | Women^1,2^(n=11905) | | |
|  | All | Diet Score of 0-3  (N=2827) | Diet Score of 4-5  (N=3893) | Diet Score of 6-9 (N=2631) |  | All | Diet Score of 0-3  (N=3970) | Diet Score of 4-5  (N=5047) |
| **Vegetables** |  |  |  |  |  |  |  |  |
| Median (g/day) | 178.2 |  |  |  | 182.4 |  |  |  |
| >Median |  | 797 (18.2) | 1957 (50.3) | 707 (26.9) |  | 1123 (28.3) | 2647 (52.5) | 2182 (75.6) |
| <Median |  | 2030 (71.8) | 1936 (49.7) | 1924 (73.1) |  | 2847 (71.7) | 2400 (47.6) | 706 (24.5) |
| **Legumes** |  |  |  |  |  |  |  |  |
| Median (g/day) | 35.7 |  |  |  | 27.5 |  |  |  |
| >Median |  | 761 (26.9) | 1961 (50.4) | 1956 (74.3) |  | 1106 (27.9) | 2607 (51.7) | 2248 (77.8) |
| <Median |  | 2066 (73.1) | 1932 (49.6) | 675 (25.7) |  | 2864 (72.1) | 2440 (48.4) | 640 (22.2) |
| **Fruits and nuts** |  |  |  |  |  |  |  |  |
| Median (g/day) | 229.7 |  |  |  | 228.9 |  |  |  |
| >Median |  | 866 (30.6) | 1956 (50.2) | 1852 (70.4) |  | 1312 (33.1) | 2604 (51.6) | 2036 (70.5) |
| <Median |  | 1961 (69.4) | 1937 (49.8) | 779 (29.6) |  | 2658 (67.0) | 2443 (48.4) | 852 (29.5) |
| **Dairy Products** |  |  |  |  |  |  |  |  |
| Median (g/day) | 159.8 |  |  |  | 138.7 |  |  |  |
| >Median |  | 1774 (62.8) | 1881 (48.3) | 1021 (38.8) |  | 2437 (61.4) | 2433 (48.2) | 1080 (37.4) |
| <Median |  | 1053 (37.3) | 2012 (51.7) | 1610 (61.2) |  | 1533 (38.6) | 2614 (51.8) | 1808 (62.6) |
| **Cereals*** |  |  |  |  |  |  |  |  |
| Median (g/day) | 130.0 |  |  |  | 113.5 |  |  |  |
| >Median |  | 1010 (35.7) | 1923 (49.4) | 1741 (66.2) |  | 1553 (39.1) | 2541 (50.4) | 1859 (64.4) |
| <Median |  | 1817 (64.3) | 1970 (50.6) | 890 (33.8) |  | 2417 (60.9) | 25063 (49.7) | 1029 (35.6) |
| *Cereals included bars power, milk rice, corn, rice, bread, bagels, biscuits, pasta, tortilla, etc.  ^1^ Values are expressed as n (%) unless otherwise specified  ^2^ Row percentages may not add to 100 due to rounding | | | | | | | | |

| **Table 1 (continued). Daily dietary, energy, and alcohol Intake by gender and Med-style diet score group** | | | | | | | | |
| --- | --- | --- | --- | --- | --- | --- | --- | --- |
| Dietary Variable |  | Men^1,2^(n=9351) | | |  | Women^1,2^(n=11905) | | |
|  | All | Diet Score of 0-3  (N=2827) | Diet Score of 4-5  (N=3893) | Diet Score of 6-9  (N=2631) | All | Diet Score of 0-3  (N=3970) | Diet Score of 4-5  (N=5047) | Diet Score of 6-9  (N=2888) |
| **Meat** |  |  |  |  |  |  |  |  |
| Median (g/day) | 91.2 |  |  |  | 69.6 |  |  |  |
| >Median |  | 1632 (57.7) | 1966 (50.5) | 1077 (40.9) |  | 2310 (58.2) | 2515 (49.8) | 1135 (39.3) |
| <Median |  | 1195 (42.3) | 1927 (49.5) | 1554 (59.1) |  | 1660 (41.8) | 2532 (50.2) | 1753 (60.7) |
| **Fish** |  |  |  |  |  |  |  |  |
| Median (g/day) | 18.6 |  |  |  | 15.9 |  |  |  |
| >Median |  | 879 (31.1) | 1971 (50.6) | 1816 (69.0) |  | 1348 (34.0) | 2568 (50.9) | 2034 (70.4) |
| <Median |  | 1948 (68.9) | 1922 (49.4) | 815 (31.0) |  | 2622 (66.1) | 2479 (49.1) | 854 (29.6) |
| **Energy Intake** |  |  |  |  |  |  |  |  |
| Median (kcal/day) | 1743.9 |  |  |  | 1460.6 |  |  |  |
| >Median |  | 1349 (47.7) | 1913 (49.1) | 1414 (53.7) |  | 1945 (49.0) | 2478 (49.1) | 1530 (53.0) |
| <Median |  | 1478 (52.3) | 1980 (50.9) | 1217 (42.3) |  | 2025 (51.0) | 2569 (50.9) | 1358 (47.0) |
| **Alcohol Intake** |  |  |  |  |  |  |  |  |
| Moderate (drinks/ week) | 1-14 |  |  |  | 1-7 |  |  |  |
| Heavy/ None |  | 816 (28.9) | 1759 (45.2) | 1685 (64.0) |  | 558 (14.1) | 1466 (29.1) | 1335 (46.2) |
| Moderate |  | 2011 (71.1) | 2134 (54.8) | 946 )36.0) |  | 3412 (85.9) | 3581 (71.0) | 1553 (53.8) |
| **Ratio of monosaturated lipids to saturated lipids** |  |  |  |  |  |  |  |  |
| Median | 1.3 |  |  |  | 1.3 |  |  |  |
| >Median |  | 650 (23.0) | 2123 (54.5) | 2242 (85.2) |  | 806 (20.3) | 2797 (55.4) | 2427 (84.0) |
| <Median |  | 2177 (77.0) | 1770 (45.5) | 389 (14.8) |  | 3164 (79.7) | 2250 (44.6) | 461 (16.0) |
| *Cereals included bars power, milk rice, corn, rice, bread, bagels, biscuits, pasta, tortilla, etc.  ^1^ Values are expressed as n (%) unless otherwise specified  ^2^ Row percentages may not add to 100 due to rounding | | | | | | | | |

Table 2. Detailed definitions for sociodemographics, health behaviors, and comorbidities.

| **Variable** | **Definition** |
| --- | --- |
|  |  |
| **Sociodemographics** |  |
| Age | Age in years |
| Gender | Male, female |
| Race | African American, white |
| Education | Participant reported:   - Less than high school - High school graduate - Some college - College or higher - Missing |
| Income | Participant reported:   - <$20k - $20k-$34k - $35k-$74k - ≥$75k - Missing (not reported) |
| Geographic Region | Participant residence:   - Stroke Buckle (coastal plains of North Carolina, South Carolina and Georgia) - Stroke Belt (remainder of North Carolina, South Carolina and Georgia, plus Tennessee, Mississippi, Alabama, Louisiana and Arkansas) - Non-Belt/Buckle (other states) |
|  |  |
| **Health Behaviors** |  |
| Current Smoker | Participant reported:   - Yes - No |
| Sedentary Behavior | Participant reported:   - 4+ hours/day of watching TV and no physical activity |
| **Comorbid Conditions** |  |
| Diabetes | Fasting glucose >126 mg/dL, non-fasting glucose >200 mg/dL, or insulin/oral hypoglycemic agent usage |
| Hypertension | Systolic blood pressure >140 mmHg, diastolic blood pressure >90mmHg, or antihypertensive agent usage |
| Stroke | Participant self-reported history of stroke |
| Atrial Fibrillation | Participant self-report or baseline electrocardiographic evidence |
| Obesity Status | BMI:   - Obese: > 30.0 - Overweight: 25.0-29.9 - Normal 18.5-24.9 - Underweight <18.5 |
